# Outcomes of Proton Beam Therapy Compared With Intensity-Modulated Radiation Therapy for Uterine Cancer

**DOI:** 10.14338/IJPT-22-00020.1

**Published:** 2022-11-14

**Authors:** Justin D. Anderson, Molly M. Voss, Brady S. Laughlin, Allison E. Garda, Khaled Aziz, Trey C. Mullikin, Michael G. Haddock, Ivy A. Petersen, Todd A. DeWees, Sujay A. Vora

**Affiliations:** 1 Department of Radiation Oncology, Mayo Clinic, Phoenix, AZ, USA; 2 Department of Health Sciences Research, Division of Biostatistics, Mayo Clinic, Scottsdale, AZ, USA; 3 Department of Radiation Oncology, Mayo Clinic, Rochester, MN, USA

**Keywords:** uterine, endometrial, proton therapy

## Abstract

**Purpose:**

To compare Patient-Reported Outcomes version of the Common Terminology Criteria for Adverse Events (PRO-CTCAE) in patients with endometrial cancer receiving adjuvant pelvic radiation therapy with proton beam therapy (PT) versus intensity-modulated radiation therapy (IMRT).

**Materials and Methods:**

Patients with uterine cancer treated with curative intent who received either adjuvant PT or IMRT between 2014 and 2020 were identified. Patients were enrolled into a prospective registry using a gynecologic-specific subset of PRO-CTCAE designed to assess symptom impact on daily living. Questions included gastrointestinal (GI) symptoms of diarrhea, flatulence, bowel incontinence, and constipation in addition to other pertinent gynecologic, urinary, and other general symptoms. Symptom-based questions were on a 0- to 4-point scale, with grade 3+ symptoms occurring frequently or almost always. Patient-reported toxicity was analyzed at baseline, end of treatment (EOT), and at 3, 6, 9, and 12 months after treatment. Unequal variance *t* tests were used to determine if treatment type was a significant factor in baseline-adjusted PRO-CTCAE.

**Results:**

Sixty-seven patients met inclusion criteria. Twenty-two received PT and 45 patients received IMRT. Brachytherapy boost was delivered in 73% of patients. Median external beam dose was 45 Gy for both PT and IMRT (range, 45-58.8 Gy). When comparing PRO-CTCAE, PT was associated with less diarrhea at EOT (*P* = .01) and at 12 months (*P* = .24) than IMRT. Loss of bowel control at 12 months was more common in patients receiving IMRT (*P* = .15). Any patient reporting grade 3+ GI toxicity was noted more frequently with IMRT (31% versus 9%, *P* = .09).

**Discussion:**

Adjuvant PT is a promising treatment for patients with uterine cancer and may reduce patient-reported GI toxicity as compared with IMRT.

## Introduction

While early-stage endometrial cancer can often be treated with surgery, adjuvant radiation therapy (RT) is considered for patients with risk factors including older age, deep myometrial invasion, higher grade, cervical stromal invasion, and lymphovascular space invasion. Radiation treatment options include external beam radiation therapy (EBRT), brachytherapy, or combination treatment [1–3]. While adjuvant RT is associated with improved local control, it is also known to cause acute and chronic gastrointestinal (GI) and genitourinary (GU) toxicity [4].

Radiation delivery techniques have evolved, allowing more sparing of surrounding normal tissues such as small bowel, rectum, bone marrow, and bladder. A clinical decrease in symptoms was first noted in the PORTEC-2 trial where vaginal brachytherapy was associated with decreased diarrhea and fecal leakage as compared with EBRT [2]. This reduction in side effects was associated with fewer limitations during daily life and better social functioning. This benefit was aided by the reduction in bowel dose with brachytherapy as compared with EBRT. RTOG 1203 also demonstrated improved GI and GU toxicity with intensity-modulated radiation therapy (IMRT) compared with standard 4-field RT [5]. Similar to brachytherapy, IMRT reduced normal tissue radiation exposure, which resulted in lower rates of diarrhea, frequency of fecal incontinence, and interference of daily life from incontinence. Dosimetric advantages with IMRT in reducing dose to small bowel, large bowel, and bladder, as compared with standard 4-field RT, are likely the cause of these improvements.

Proton therapy (PT) has the potential to provide further dosimetric advantages over IMRT, including decreased dose to pelvic normal structures. Proton therapy can better spare the small intestine, colon, bilateral femoral heads, bladder, and pelvic bones than both IMRT and 3D RT [6, 7]. Additional data are needed to show if the improved dosimetry of PT is associated with clinical benefits and decreased symptoms. The purpose of our study is to compare prospectively gathered patient-reported outcomes (PROs) for patients receiving adjuvant PT or IMRT for uterine cancer at a multisite institution.

## Materials and Methods

Institutional review board approval was received to proceed with a retrospective review of a prospectively collected database for patients with uterine cancer at a multisite institution. The database included patient demographics, treatment-related information, and PROs. Between 2014 and 2020, patients with endometrial cancer who were treated with surgery followed by adjuvant pelvic RT with or without para-aortic EBRT were included in this study. Patient characteristics and treatment information not available or missing from the database were collected from the medical record.

### Simulation and Treatment Planning

All patients underwent computed tomography–based simulation with and without intravenous contrast. Patients receiving PT had carbon fiber markers placed at the vaginal cuff and a rectal balloon inserted. Patients were simulated in the supine position with custom pelvic immobilization. For patients treated with protons, simulation occurred with an empty bladder. Oral, rectal, and vaginal contrast were avoided in patients treated with PT to identify true Hounsfield units for treatment planning purposes. For patients treated with IMRT, simulation occurred with both full and empty bladder scans and treatment was typically delivered with a full bladder.

Nodal contours followed RTOG gynecologic contouring guidelines, which included identification of nodal regions including external iliac, internal iliac, presacral, obturator, and para-aortic regions as clinically indicated. A 5- to 7-mm margin was added to the nodal clinical target volume (CTV) to create a nodal planning target volume [8]. The vaginal CTV contours included the vagina and paravaginal soft tissue with 1-cm lateral expansion. A vaginal cuff internal target volume (ITV) was created from full and empty bladder scans for patients treated with IMRT. The vaginal/parametrial ITV was expanded 0.5 cm isometrically to create the planning target volume for patients treated with IMRT and 1 cm laterally and inferiorly and 0.5-cm expansion anteriorly, posteriorly, and superiorly to create the optimization target volume (OTV) for patients treated with PT. Vaginal brachytherapy and simultaneously integrated boosts to clinically present nodal disease were delivered when clinically indicated at the discretion of the treating physician.

Proton therapy treatment planning was performed by using our institution's individual field simultaneous optimization technique. This technique has been previously described for patients with head and neck cancer [10]. In brief, CTVs were divided into subtargets to allow for the creation of individual OTVs. This method allowed the beam to avoid traveling through heterogeneous mediums such as bowel while keeping the mean range error small. An additional scanning target volume was then created to demarcate the area where spots could be placed to adequately target the OTV. Scanning target volumes are an expansion off the OTV to attain lateral equilibrium due to the Gaussian-like dose falloff from the proton spots. Separate OTVs could either be targeted by just 1 or multiple beams to improve plan conformity. A typical beam arrangement consisted of a posterior beam to treat the nodal chains and slightly anterior oblique beams to treat the vaginal/parametrial targets.

### Patient-Reported Outcomes

Patients were given a gynecologic-specific subset of Patient-Reported Outcomes version of the Common Terminology Criteria for Adverse Events (PRO-CTCAE; US National Cancer Institute, Bethesda, Maryland) designed to assess symptom impact on daily living. Surveys were distributed at baseline before radiation start with additional follow-up administered at end of treatment (EOT), and at 3, 6, and 12 months after treatment. Gastrointestinal questions included symptoms of diarrhea, flatulence, bowel incontinence, and constipation. Patients were also asked questions about sexual health, GU symptoms, and extremity swelling. Symptom-based questions consisted of a 5-point Likert scale that was converted to a grade of 0 to 4 for statistical analysis. A score of 0 indicated not at all or never, 1 indicated a little bit or rarely, 2 indicated somewhat or occasional, 3 indicated quite a bit or frequently, and 4 indicated very much or almost constantly.

### Statistical Analysis

The events of interest were PRO-CTCAE survey results after radiation treatment following the standard-of-care timepoints (EOT, 3 months, 6 months, yearly). Summary statistics were reported for patient characteristics, pathology, treatment, and dosimetry data and are reported as mean, standard deviation and range or percentage (N) as appropriate. Kruskal-Wallis and Fischer exact tests were used to compare treatment modalities as appropriate. PRO-CTCAE events were predicted with logistic regression and reported with odds ratio (95% confidence interval). Statistical significance was defined as *P* < .05. Statistical analyses were conducted with R version 3.4.0 (R Core Team, 2014) and figures were produced with the ggplot2 package (Wickham, 2009).

## Results

Sixty-seven patients were included in this analysis. The median follow-up was 2.2 years (interquartile range [IQR], 1.0-2.8 years) for patients receiving PT and 2.8 years (IQR, 0.7-4.0 years) for patients treated with photons. Patient characteristics are listed in Table 1. The median age at the start of RT was 67 years old. Most patients were white (85%). The median body mass index (BMI) was 31 kg/m^2^, with patients treated with protons having a smaller BMI than those treated with photons (28 versus 35, *P* = .001).

**Table 1.** Patient characteristics.

**Patient characteristics**	**Proton (N = 22)**	**IMRT (N = 45)**	**Total (N = 67)**	***P*** **value**
Age, y				<.001
Mean (SD)	72.7 (6.1)	64.8 (8.8)	67.4 (8.8)	
Range	61–83	38–87	38–87	
Race, n (%)				.6
White	18 (81.8)	39 (86.7)	57 (85.1)	
Other	4 (18.2)	6 (13.3)	10 (14.9)	
BMI, kg/m^2^				.001
Mean (SD)	28.0 (5.2)	34.9 (9.1)	32.6 (8.6)	
Range	19.9–38.4	18.6–57.9	18.6–57.9	
Tobacco, n (%)				.40
No	15 (68.2)	35 (77.8)	50 (74.6)	
Former	7 (31.8)	10 (22.2)	17 (25.4)	
Histology, n (%)				.99
Endometrioid	12 (54.5)	24 (53.3)	36 (53.7)	
Carcinosarcoma	4 (18.2)	8 (17.8)	12 (17.9)	
Clear cell	1 (4.5)	4 (8.9)	5 (7.5)	
Serous	5 (22.7)	9 (20.0)	14 (20.9)	
Grade, n (%)				.82
1 (well differentiated)	4 (18.2)	5 (11.4)	9 (13.6)	
2 (moderately differentiated)	6 (27.3)	14 (31.8)	20 (30.3)	
3 (poorly differentiated)	11 (50.0)	25 (56.8)	36 (54.5)	
4 (undifferentiated)	1 (4.5)	0 (0.0)	1 (1.5)	
missing	0	1	1	
Stage, n (%)				.02
I	9 (40.9)	7 (15.6)	16 (23.9)	
II	2 (9.1)	2 (4.4)	4 (6.0)	
III	10 (45.5)	35 (77.8)	45 (67.2)	
IV	1 (4.5)	1 (2.2)	2 (3.0)	
Recurrence, n (%)				.03
No	19 (86.4)	45 (100.0)	64 (95.5)	
Yes	3 (13.6)	0 (0.0)	3 (4.5)	

**Abbreviations:** IMRT, intensity-modulated radiation therapy; BMI, body mass index.

Twenty-two patients were treated with PT and 45 were treated with IMRT. For patients treated with PT, most were treated in the primary setting after surgery (86%). The remaining 3 patients (14%) were treated for recurrent disease without history of prior RT. All patients receiving IMRT were treated in the primary setting after surgery. There was a lower proportion of stage III patients in the proton group than photon group, 46% versus 78% (*P* = .01), respectively. Vaginal brachytherapy boost was delivered in 76% of patients treated with protons and in 78% of those treated with photons. Median brachytherapy boost dose was different between the 2 groups owing to institutional differences in practice patterns. Median brachytherapy dose in patients receiving PT was 15 Gy in 3 fractions compared with 10 Gy in 2 fractions for patients treated with photons. Para-aortic fields were included for 14% of patients receiving PT compared with 33% receiving photon therapy (*P* = .14). Concurrent chemotherapy was only delivered in 2 patients, both treated with IMRT. Treatment characteristics are listed in **Table 2**.

**Table 2.** Treatment characteristics.

**Treatment characteristics**	**Protons (N = 22)**	**IMRT (N = 45)**	**Total (N = 67)**	***P*** **value**
Chemo before RT, n (%)				.07
No	9 (40.9)	9 (20.0)	18 (26.9)	
Yes	13 (59.1)	36 (80.0)	49 (73.1)	
Chemo after RT, n (%)				.11
No	17 (77.3)	25 (55.6)	42 (62.7)	
Yes	5 (22.7)	20 (44.4)	25 (37.3)	
EBRT dose to primary, Gy				.88
Median	45	45	45	
Range	45–50.4	45–50	45–50.4	
HDR boost, n (%)				.02
No	2 (9.1)	16 (35.6)	17 (25.4)	
Yes	20 (90.9)	29 (64.4)	50 (74.6)	
Boost dose, Gy				<.001
Median	15	10	10	
Range	10–25	10–20	10–25	
PA nodes, n (%)				.14
No	19 (86.4)	30 (66.7)	49 (73.1)	
Yes	3 (13.6)	15 (33.3)	18 (26.9)	

**Abbreviations:** IMRT, intensity-modulated radiation therapy; RT, radiation therapy; EBRT, external beam radiation therapy; HDR, high dose-rate; PA, para-aortic.

**Figure 1.**
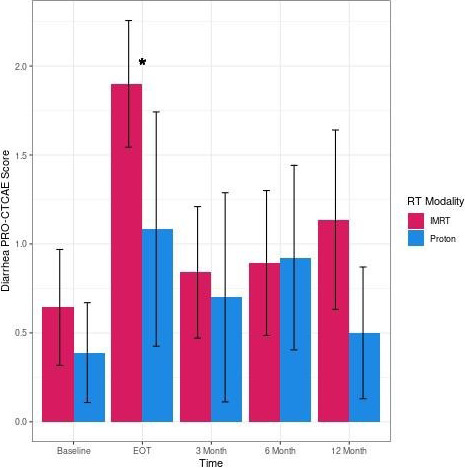
Diarrhea PRO-CTCAE scores of patients receiving proton therapy compared with patients receiving IMRT at different time points. *P value <.05. Abbreviation: EOT, end of treatment; IMRT, intensity-modulated radiation therapy; PRO-CTCAE, Patient-Reported Outcomes version of the Common Terminology Criteria for Adverse Events; RT, radiation therapy.

Survey completion rate at EOT, and 3, 6, and 12 months after radiation was 67%, 56%, 62%, and 49%, respectively, for patients receiving IMRT and 55%, 45%, 59%, and 36%, respectively, for patients receiving PT. Pertinent PRO-CTCAE adverse events comparisons are as follows: Any grade >2 PRO-CTCAE was reported in 82% of PT patients and 91% of IMRT patients (*P* = .28), and any grade >3 PRO-CTCAE was reported in 77% of PT patients and 87% of IMRT patients (*P* = .33). Any GU grade >2 PRO-CTCAE was reported in 64% of PT patients and 73% of IMRT patients (*P* = .43), and any GU grade >3 PRO-CTCAE was reported in 23% of PT patients and 36% of IMRT patients (*P* = .25). Any diarrhea grade >2 PRO-CTCAE was reported in 46% of PT patients and 67% of IMRT patients (*P* = .10), and any diarrhea grade >3 PRO-CTCAE was reported in 9% of PT patients and 33% of IMRT patients (*P* = .05). **Figure 1** shows the difference in average diarrhea PRO-CTCAE score over time.

**Figure 2.**
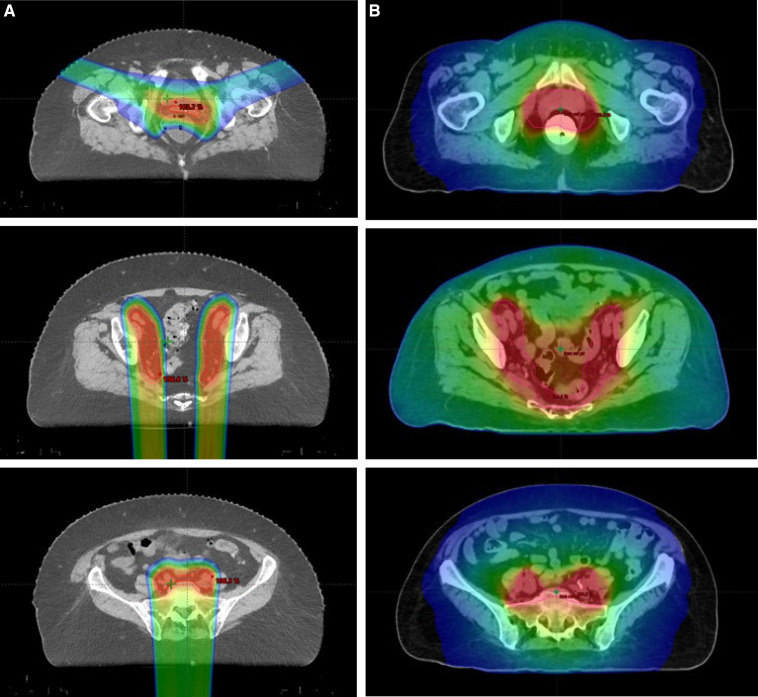
Dose color wash for (A) a proton plan versus (B) an IMRT plan. Abbreviation: IMRT, intensity-modulated radiation therapy.

The biggest differences between doses delivered to organs at risk were noted in low to intermediate dose ranges. Proton therapy resulted in significantly improved small bowel V15 (125 cm^3^ versus 856 cm^3^, *P* < .001) and V30 (90 cm^3^ versus 263 cm^3^, *P* < .001), bladder V45 (28% versus 34%, *P* = .01), bone marrow V10 (59% versus 84%, *P* < .001) and V20 (49% versus 76%, *P* < .001), and rectum V30 (36% versus 75%, *P* < .001) and V40 (27% versus 54%, *P* < .001). There was no difference when comparing small bowel V45 (cm^3^) and V40 (%). Logistic regression of the dose-volume histogram and PRO-CTCAE scores showed no correlation between dose to organs at risk and increased PRO-CTCAE toxicity. Computed tomography scans with the accompanying dose color wash from external beam radiation are listed in **Figure 2**.

## Discussion

Proton therapy for endometrial cancer is an emerging and promising treatment option in the adjuvant setting to maximize clinical benefit and minimize treatment morbidity. Our data show the potential for decreased GI toxicity with PT compared with IMRT. Patients treated with PT reported a nearly 1-point reduction in diarrhea frequency, compared with IMRT, at EOT (1.1 versus 1.9). This difference would represent rarely experiencing diarrhea compared with occasionally experiencing diarrhea. Additionally, 23% of patients treated with IMRT reported grade >2 loss of bowel control 12 months after radiation, compared with 0 patients treated with PT.

Reducing GI and GU toxicity is an important goal for patients with uterine malignancy. A quality-of-life analysis of PORTEC-2 showed EBRT significantly increased rates of diarrhea and fecal leakage while also negatively impacting social functioning.^2^ GOG 249, a more recently published randomized controlled trial for early high-intermediate risk endometrial cancer, still reported 44% grade 2 or greater adverse events in patients who received pelvic radiation despite allowing the use of IMRT [10]. The impact of adverse events related to RT has been shown to negatively impact vitality and physical and social well-being [11].

Significant efforts have been made to improve outcomes and reduce toxicity for patients with uterine and pelvic cancer. The results of RTOG 1203 demonstrated a significant reduction in patient-reported acute GI and GU toxicity for IMRT compared with 3D-conformal RT.^5^ Patients treated with IMRT experienced less acute GI toxicity at week 5 during radiation. Urinary toxicity was also less with IMRT. Another phase 3 trial comparing 3D-conformal RT with image-guided IMRT for adjuvant cervical cancer showed decreased 4-year grade >2 and grade >3 GI toxicity with image-guided IMRT. The reduction in GI toxicity in this trial was correlated with lower V30 and V40 bowel bag doses with image-guided IMRT [12]. Despite the recent improvements with IMRT, improved quality of life and reduced treatment-related adverse events are still an important goal.

Given the reduced toxicity with improvements in image guidance, treatment planning, and treatment delivery, PT is another promising tool to further reduce acute and late toxicity for patients with uterine cancer. Proton therapy has the potential to reduce adverse events while maintaining equivalent cure rates. This is due to the decreased integral dose and improved dose conformality around the treatment target. Our data support previously published data showing the ability of PT to further improve dosimetry, compared with IMRT, especially in low-dose regions [7]. Dosimetric studies have shown PT can reduce the dose of radiation to the small bowel, large bowel, pelvic bone marrow, and bladder, especially in low- and intermediate-dose levels [6, 7]. The impact of this improved dosimetry requires further investigation to determine the clinical significance of these findings. Current phase 2 prospective studies are evaluating if this dosimetric improvement results in improved quality of life and treatment outcomes for patients with uterine cancer [13, 14].

This study has many limitations. The sample size of this study came from only 2 institutions and was relatively small, with modest survey response rates. While there were no significant differences in baseline GI and GU toxicity rates between patients receiving PT and IMRT, there were significant differences in some patient characteristics including age, BMI, and stage at diagnosis. Differences in treatment characteristics such as rate of para-aortic fields treated with PT and IMRT may have also impacted rates of adverse events. Even though PT resulted in improved dose-volume histogram findings, the dosimetric differences did not correlate with statistically significant rates or grade >2 or grade >3 adverse events. This is likely influenced by the low number of patients included in this study.

## Conclusion

Proton therapy is a promising treatment technique that may help further reduce acute and potentially late GI and GU side effects. Our study demonstrated the safety and utility of PT in uterine cancer. Our data show PT may decrease the frequency of diarrhea at the end of radiation therapy and lower the risk of bowel incontinence at 12 months as compared with IMRT techniques for this patient population. Further investigation is needed to define the role and benefit of PT for uterine cancer.
